# Intensive care unit nurses' perceptions of patient participation in the acute phase of chronic obstructive pulmonary disease exacerbation: an interview study

**DOI:** 10.1111/j.1365-2648.2012.06021.x

**Published:** 2013-02

**Authors:** Marit Kvangarsnes, Henny Torheim, Torstein Hole, Lennart S Öhlund

**Affiliations:** Aalesund University CollegeNorway; Helse Møre og Romsdal, Aalesund University CollegeNorway; Medical Clinic, Helse Møre og RomsdalNorway and Medical Faculty, Norwegian University of Science and Technology (NTNU)Trondheim, Norway; Aalesund University CollegeNorway

**Keywords:** COPD exacerbation, discourse analysis, nurses, patient participation

## Abstract

**Aim:**

To report a study conducted to explore intensive care unit nurses’ perceptions of patient participation in the acute phase of chronic obstructive pulmonary disease exacerbation.

**Background:**

An acute exacerbation is a life-threatening situation, which patients often consider to be extremely frightening. Healthcare personnel exercise considerable power in this situation, which challenges general professional notions of patient participation.

**Design:**

Critical discourse analysis.

**Methods:**

In the autumn of 2009, three focus group interviews with experienced intensive care nurses were conducted at two hospitals in western Norway. Two groups had six participants each, and one group had five (*N* = 17). The transcribed interviews were analysed by means of critical discourse analysis.

**Findings:**

The intensive care nurses said that an exacerbation is often an extreme situation in which healthcare personnel are exercising a high degree of control and power over patients. Patient participation during exacerbation often takes the form of non-involvement. The participating nurses attached great importance to taking a sensitive approach when meeting patients. The nurses experienced challenging ethical dilemmas.

**Conclusion:**

This study shows that patient participation should not be understood in universal terms, but rather in relation to a specific setting and the interactions that occur in this setting. Healthcare personnel must develop skill, understanding, and competence to meet these challenging ethical dilemmas. A collaborative inter-professional approach between physicians and nurses is needed to meet the patients’ demand for involvement.

## Introduction

Patient participation is a predominant issue in healthcare discourses in many countries and health systems worldwide (Florin *et al.* 2006, Thompson 2007, Sahlsten *et al.* 2008, Aasen *et al.* 2011), and nurses’ perceptions of chronic obstructive pulmonary disease (COPD) treatment can be used to explore further the notions of patient participation. An acute exacerbation of COPD is a life-threatening condition, and patients often experience this situation to be extremely frightening (Bailey 2001, Torheim & Gjengedal 2010). Patients with acute exacerbation are in some parts of Norway admitted to intensive care units where they undergo non-invasive positive-pressure ventilation via bilevel positive airway pressure (BiPAP) ventilation with mask (Keenan *et al.* 2003, Ambrosino & Vagheggini 2007). Patients are in a vulnerable phase of life, and healthcare personnel occupy a complementary position of power. According to the Norwegian Patients’ Rights Act (1999), patients in Norway have the right to participate in their treatment. As nurses are involved in managing the prescribed treatment, they have a role in ensuring that patients’ rights are fulfilled.

### Background

Patient participation has gained increasing prominence in healthcare policies. Policy documents highlight the need for patient involvement at all levels of treatment [White paper no. 25 2005–2006, World Health Organization (WHO) 1994, 2005]. Patient participation is advocated on the grounds that it will lead to an array of benefits such as improved patient satisfaction, greater patient cooperation with healthcare professionals, better management of disease, increased trust, and enhanced patient–professional relationships (Collins *et al.* 2007).

However, the concept of patient participation is complex and multifactorial in nature (Cahill 1996, Collins *et al.* 2007). Participation is understood as ‘being informed’, and it manifests itself through partaking in decision-making about care and treatment (Eldh 2006). Researchers (Eldh *et al.* 2004, Eldh 2006) have argued that patient participation implies not only having knowledge about body, illness, and symptoms but also providing information to healthcare staff, being listened to, trusted, and recognized for one’s knowledge and experience, and being considered a resourceful individual.

Patient participation can be seen as twofold because the participating partners in clinical interactions – the healthcare giver and the healthcare receiver – must have a common understanding and respect for each other’s contribution (Eldh 2006). Sahlsten *et al.* (2008, p. 9) explained patient participation in nursing practice as follows: ‘Patient participation in nursing practice can be defined as an established relationship between nurse and patient, a surrendering of some power or control by the nurse, shared information and knowledge, and active engagement together in intellectual and/or physical activities’. Sahlsten *et al.* (2005) investigated obstacles to patient participation in somatic care and identified nurses’ paternalistic attitude and lack of insight and knowledge as factors that may impede patient participation. Other factors included the influence of significant others and organization and work environment (Sahlsten *et al.* 2005).

Thompson *et al.* (2007, p. 192–193) constructed a framework for facilitating future development in practice, teaching, and research about patient participation and made the following recommendations:

- Participation can be understood in terms of components, levels, and contexts.
- A distinction should be made between desired and achieved levels of participation.
- Participation cannot be understood in global terms but only in relation to specific settings and types of consultations.
- Attempts to measure patient participation or evaluate its outcomes need to begin with, and remain sensitive to, an understanding of the contextual influence of its forms.

An overview of the literature exploring the care needs of patients with advanced COPD indicated that they have a high symptom burden, which impairs their quality of life and social functioning. Patients and their families seldom have a complete understanding of the condition and its prognosis, and information needs in COPD are often unmet. Some obstacles to the provision of palliative care to patients with COPD include a professional reluctance to negotiate end-of-life discussions and a perceived lack of understanding amongst patients regarding illness trajectory (Gardiner *et al.* 2010).

## The study

### Aim

The aim of the study was to explore intensive care unit nurses’ perceptions of patient participation in the acute phase of COPD exacerbation. An analysis of nurses’ accounts of these acute situations may shed greater light on the basic and complex issues related to patient participation.

### Design

Critical Discourse Analysis (CDA) was used as a theoretical framework and a methodological orientation (Fairclough 1992, 1995, 2001). Data were collected through focus group interviews with experienced intensive care nurses (McLafferty 2004, Barbour 2005).

### Theoretical framework

We chose CDA as the most appropriate method. CDA is an analytical framework for researching language in relation to power and ideology, which brings ‘together linguistically oriented discourse analysis and social and political thought relevant to discourse and language’ (Fairclough 1992, p. 62). Fairclough (2001, p. 91) created a three-dimensional framework for discourse analysis in which three stages of discourse analysis are distinguished: (i) description of text, (ii) interpretation of the relationship between text and interaction, and (iii) explanation of the relationship between interaction and social context. Explanation is a matter of viewing a discourse as part of social struggle, within a matrix of power relations (Fairclough 2001, p. 135).

In the present study, Fairclough’s analytical levels of description and interpretation were emphasized. The focus group interviews, which were viewed as discursive events, were analysed as text and discursive practice. Fairclough (1995, p. 9) stated that ‘the interpretation of texts is a dialectical process resulting from the interface of the variable interpretative resources people bring to bear on the text, *and* properties of the text itself’. In the present study, theory on patient participation (Thompson *et al.* 2007) and CDA comprised a framework to explain the focus group interviews as social practice.

### Participants

We used purposive sampling to recruit participants for this study. The participants were chosen because they knew much about the topic. The inclusion criteria were as follows: (i) advanced education in intensive care nursing and (ii) at least 2 years of experience in treating COPD. Two of the focus groups consisted of nurses who had been recruited from a city hospital, whereas the third group comprised nurses from a smaller hospital in a rural district. The groups were interviewed at two hospitals in western Norway in autumn 2009. Emphasis was placed on ensuring that no conflict of interest existed amongst the participants in each group. One of the researchers recruited informants by inviting them face-to-face during work hours. Two of the nurses who had been invited to participate declined for practical reasons. The study included 17 participants who were divided into three groups: two groups with six participants each and one group with five participants. All of the participants were Norwegian women between 41–62 years and had more than 10 years of nursing experience.

### Data collection

Focus group interviews were conducted with participating nurses in hospital meeting rooms. At the time of interview, the nurses had no clinical obligations. The nurses in each focus group knew and appeared to trust one another. During the interviews, the nurses spontaneously recounted their experiences and offered their reflections.

We developed an interview guide with open questions that focused on the nurses’ clinical experiences and their perceptions of patient participation during the acute phase of COPD exacerbation. The basis for producing the interview guide was earlier research (Eldh 2006, Thompson *et al.* 2007, Sahlsten *et al.* 2008) and its theories on patient participation and the aim of the study. The interview guide was also discussed with experienced intensive care nurses. Two experienced intensive care nurses with formal interview research training moderated the focus groups. The moderators were two of the authors and were not colleagues of the participants. One of the moderators guided the discussion according to the interview guide (Polit & Beck 2008). The participating nurses were asked to elaborate on their clinical experiences, perceptions of patient participation, and the factors that facilitate and inhibit it. The interviews, which lasted between 80–90 minutes, were audio-recorded and then transcribed verbatim. In addition, a second moderator took field notes during the interviews. At the end of each interview, the second moderator summarized what had been said, and the group members responded to this summary. All of the informants stated that the summaries were in accordance with what they had tried to convey. After the third interview and preliminary readings, data were found to be rich and thick (Morse & Richards 2002). The data showed the complexity in the intensive care unit nurses’ perceptions of patient participation in the acute phase of COPD exacerbation. The researchers got a feeling of having heard it all. Data replicated and we concluded that information from the empirical data was sufficiently saturated.

### Ethical considerations

This study was a part of a larger research project approved by the regional committee for health research ethics (4.2008.2869). The nurses were told that participation was voluntary and that their anonymity would be protected. The researchers also made it clear that the nurses could withdraw from the study at any time. The participants received both written and oral information, and they gave their informed consent. All the focus groups seemed to trust the researchers and shared information about difficult ethical situations. The researchers emphasized discussing methods of enhancing the trustworthiness of the study’s data (Polit & Beck 2008).

### Data analysis

Four researchers read the transcribed interviews, giving special attention to identifying words, expressions, and phrases that characterized the discourse of patient participation. Samples from all of the interviews were selected for detailed analysis based on the following criteria: The samples illustrated the nurses’ perceptions of patient participation; offered a range of views, thus showing the complexity of patient participation; showed what issues/topics were mentioned in the interview responses; and represented ‘a moment of crises’ (Fairclough 1992, p. 230). The criteria were chosen to find the patterns in the patient participation discourse in the data. To show some of the dynamics involved in producing the text, we present some interview excerpts in this article.

Text analysis was centred on vocabulary, grammar, coherence, and text structure. Description implied linguistic analysis. The analysis of discursive practice focused on (i) what kinds of speech acts were used, (ii) text coherence, and (iii) intertextuality (Fairclough 1992, p. 75).

### Rigour

To enhance the study′s trustworthiness, the researchers′ decisions throughout the study are carefully and explicitly described. The researchers′ relationships with the informants are also made clear. The congruence, i.e. the interconnectedness between parts of the inquiry and the whole, and between study findings and external contexts (Polit & Beck 2008) was discussed throughout the research process. The interpretation of the data was discussed with all the authors and was confirmed by all of them.

## Findings

Our analysis of the focus group interviews conducted with experienced intensive care nurses identified several typical patterns and structures. An interpretation of the relationship between text and interaction revealed three major themes: (i) low level of patient power and participation, (ii) emotional reciprocity, and (iii) patient participation in life or death situations. In the ‘Discussion’ section of this article, these three themes are examined in the context of the participation discourse as expressed in public documents and legislation. The relationship between the discursive processes and social processes is also explained.

### Low level of patient power and participation

In all three focus groups, the nurses stated that dyspnoea dominated the acute phase of COPD exacerbation in patients. The nurses also used charged words and terms, such as ‘sense of suffocation, choking, hunger for air, fear of death, and claustrophobia’, to describe how they perceived their patients’ experiences. In the following interview excerpt (interview 2), the nurses describe how patients in the acute phase of COPD exacerbation react:

Moderator (M): Is there a big difference between the patients in terms of how they react to acute exacerbation?

Nurse (N)9: Their anxiety is very deep-seated. I think that is common for all of them. Their fear is always present.

N8: We may appear to be dangerous and threatening to them – even if we only try to help.

N11: Almost like an assault.

N9: We come, and then we just do something with them.

N7: Yes, yes.

N8: Even if we try to explain it, we explain and we explain over and over again. And then they still don’t get it, or they don’t want to understand it. It’s not easy.

One nurse’s (N9) remark about the patients’ fear triggered supplementary responses from the other group members who then described similar experiences. In addition, the nurses revealed that the patients sometimes perceived the nurses as threatening because they did not understand what was happening to them. The nurses also used words such as ‘dangerous’ and ‘assault’ to describe the patients’ reactions to treatment administered by them. They often found it challenging to get patients in the acute phase of COPD exacerbation to participate in their treatment. The nurses’ use of repetitive phrases and the present tense (i.e. ‘we explain and we explain’) emphasize this point. In the following interview excerpt (interview 3), the nurses discuss what usually happens when patients are not able to participate:

N13: We never force a patient. In such cases, you must call a physician.

N17: It happens that you have to use a lot of persuasion. Sometimes I’ve felt that this has been next to using force.

N14: Actually, in a somatic care unit, you are from this year onwards allowed to use force for a short period during treatment.

N17: Hypoxic, those that are not in their full senses.

N14: You have to use force because they don’t know what’s best for them.

N17: And what we are talking about here is usually of short duration.

N14: You just have to do it to save their lives.

Some key words in this interview excerpt are ‘force’ and ‘persuasion’, which were used almost synonymously in this context. Here, the word ‘force’ is a strongly charged term. This issue was introduced when one of the informants asserted that they never use force. The next speaker modified this assertion, stating that they have to use ‘a lot of persuasion’ and characterizing this action as ‘next to using force’. One of the nurses referred to the existence of a law that legitimizes the use of force in certain situations. Hypoxia and fear inhibit patients from participating in their treatment. The assertion that ‘they don’t know what’s best for them’ legitimized the administration of treatment and the use of force. All of the focus groups explained that when life-threatening situations arise, healthcare personnel have to take control, which might entail the use of force. They also said that they had good experiences with patients receiving sedation in the acute phase of COPD exacerbation. This step is considered necessary for administering the appropriate treatment.

The nurses also encountered patients whom they thought coped well with mask treatment and participated satisfactorily in their treatment. One nurse described a patient who always made a special effort to obtain the necessary dampness/moisture during mask treatment.

The nurses in one of the focus groups emphasized the importance of reading body language. One nurse commented, ‘I can tell from his respiration that he is breathing unnecessarily quickly – not only to ventilate PCO_2_ but [also] because he is afraid. You just have to read nuances like this’. What is highlighted here is a more indirect form of participation. The nurses had a very clear point of view: In these situations, it is fundamental to understand and interpret nuances in body language so as to meet the needs of the patient. The nurses also drew on past experience to give patients the kind of nursing that they thought their patients wanted. Another indirect way of practising patient participation is by communicating with the next of kin. The nurses said that next of kin often gives a lot of information about the patient, and in this way the patients participate indirectly. All of the focus groups agreed that patient participation is far easier to achieve if the mask treatment is successful and the patient is recovering. The groups specified several areas of patient participation, including time and extent of care, meals, time and extent of activity and training, time and extent of visits, and the administration of medical treatment.

### Emotional reciprocity

All of the groups pointed out the importance of helping patients cope with their anxiety and fear and of cultivating a sense of safety in the acute phase of COPD exacerbation. In the following interview excerpt (interview 1), the nurses discuss what is most important when meeting with a patient:

M: What is important when meeting with a patient?

N6: To be calm.

N3: Don’t press on the mask right away. Give them some time.

N1: Simple information. Don’t talk too much, only enough to make them understand what is going on.

N2: But information is very basic.

N3: That’s important.

N2: Yes.

N1: But don’t ask too many questions where they have to use a lot of words to explain and answer because they can’t breathe properly. Preferably yes-or-no questions.

N3: At the same time, they can be under so much stress that they are not able to take in the information. And then I for one experience that it is very important that I have eye contact so I can be sure of having contact there and then.

All of the nurses gave further information on what factors are vital in patient participation during the acute phase of COPD exacerbation. Basic information, being calm, and keeping eye contact were considered important. One of the nurses emphasized the importance of being aware of one’s own conduct and bedside manner. All of the groups stressed how imperative it was for patients to experience their situation as being under control. This realization points to the importance of nurses assuming a professional responsibility. In the following interview excerpt (interview 1), the nurses describe how they try to convey to patients that the situation is under control:

N3: Up to a limit, we let him [the patient] keep a sense of control and … decide for himself, but in such a way that we at the same time let him know that we actually know a bit more than he does – even if he is himself and his body reminds him of it all the time. We are in a way saying, ‘OK, we do it like this and like that, and just now we are going to do it exactly like this’. You try to make a deal with the patient: First we do it like this for a while, and then we do something else for a while, all divided into defined limits of time.

N1: I think all this [discussion with patients] about making deals is very necessary – at least when they are in their [full] senses and have a sense of time because then they get very occupied with their watches and look at them all the time.

During this part of the focus group interview, the nurses described their approach to meeting with patients. The nurses’ use of phrases such as ‘let him [the patient] keep a sense of control’ and ‘making deals is very necessary’ shows that they are engaged in achieving emotional reciprocity while acting with authority. They also explained how they try to balance patient participation with mask treatment, for example, by letting the patients have the necessary pauses. To take control in such situations, the nurses use the present continuous verb tense and convey a strong modality: ‘We are going to do it exactly like this’. The nurses also conveyed how important it was to keep promises and not leaving the patient in the acute phase.

### Patient participation in life or death situations

All of the focus groups considered patient participation in life or death situations to be especially challenging. When patients reach the final stage of the disease, various situations arise. Dealing with these situations becomes difficult when physicians and patients have not adequately discussed treatment options and made decisions accordingly. In the following interview excerpt (interview 3), the nurses describe this dilemma:

N16: What is really hard is this: The patients who that have reached the final stage, Gold 4, and who have been in and out of hospital, and it turns out that it has not been decided whether the patient shall be put on a ventilator or not. And then you find yourself in this situation at 4 in the morning, and nobody has decided anything, and then you have to put the patient on [a ventilator] because you really don’t have a choice. And on top of all – it may not even be the right thing to do.

N15: And then you know that this patient will perhaps never be off the ventilator.

N16: This is frustrating. If you push press a person’s head under water and ask them if they want to drown, no one will tell you yes. And so you do not ask a patient in the acute phase if he or she—the next time it happens—want to be on a ventilator or not.

Ethically difficult care situations are discussed in this interview excerpt. The nurses acknowledged their uncertainty about the benefit of attaching a patient to a ventilator when the disease has reached a certain stage. The challenge posed to nurses is illustrated through the use of a drowning metaphor and emotionally charged words. In the following interview excerpt (interview 2), the nurses describe situations in which they had arranged a dignified end of life for their patients:

N7: I have experienced it, but as a wish expressed in a calm and quiet phase [of the disease].

N8: So, then it was palliative and not curative treatment?

N7: Yes, and it was all very fine and very dignified, with the family all around and a grandchild [performing a] song. And it all turned out to be a fine way of ending life and, in fact, a good memory for the family in the time after.

N10: Yes, but it is so important that this is clarified in advance.

N7: Yes.

N9: Yes, and that it is actually patient participation.

In this interview excerpt, the nurses used positively charged words and expressions to characterize the end of life. The nurses in all of the focus groups shared their views on how a dignified death could be arranged for patients. All agreed on the importance of removing the mask during this final phase of life and replacing it with nose spectacles. Having the next of kin present was also seen as a benefit. One of the nurses (N8) commented that she felt it to be a defeat if a patient died with the mask on (interview 2). The other nurses in the group nodded in agreement with her comment. They all seemed to consider the mask as an obstacle to a reasonable form of contact between patients and their next of kin in such situations.

This last phase of life will naturally be difficult to handle – even in those cases where patients have made it clear to their physicians during a calm period in the course of their illness that they do not want further treatment. This topic is discussed in the following interview excerpt (interview 2):

N9: There was a woman once … [who declared that she was] not going to have the ventilator, but when she got intense air hunger, she became so desperate for air, that the only thing she could say was ‘breathing machine’ [ventilator]. She died. I have never forgotten her. From the patient’s point of view, the situation may change. It is never a clear-cut case. You can change your mind.

N8: Yes, that must be OK.

N9: There are lots of dilemmas here. It is not like this or like that.

N7: In calm periods, things get clearer, and you can talk things through in a proper way and preferably repeat it.

In this interview excerpt, one of the nurses (N9) described how a patient close to death suddenly expressed new opinions about her treatment that contradicted what she had previously said. All of the focus groups pointed out that the acute phase of COPD is often an ethically demanding situation and that this phase is not a good time to raise questions concerning palliative treatment. The nurses expressed that care in these situations can be very difficult, especially when no experienced doctors who know the patients are present.

## Discussion

### Discussion of findings

The nurses who participated in this study experienced that patients suffering from COPD exacerbation often exhibit low levels of power and participation in their treatment and typically encounter ethically challenging situations. For these reasons, the nurses attached great importance to taking a sensitive approach when meeting patients.

The nurses stated that patient participation often takes the form of non-involvement under these circumstances. Because patients are often incapable of expressing themselves verbally, they are not able to take part in the reasoning process or have an influence on problem definition. To take a share in reasoning processes, to have influence in problem definition and decision-making and take part in action sequences, are components in Thompson *et al.* (2007) holistic framework for understanding patient participation. The nurses acknowledged feeling a huge professional responsibility in administering treatment. Their actions in situations like these are formally legitimized by law (viz. § 7 in the chapter on emergency health care in the Health Personnel Act, 1999). From the nurses’ point of view, treatment in the acute phase of COPD exacerbation is, to a high degree, professionally determined. Thompson *et al.* (2007) also have found that involvement might be more problematic for those patients who might be in need of emergency care rather than more routine forms of health care.

In 2009, a new paragraph was added to the Norwegian Patients’ Rights Act, 4A (Board of Health Supervision 2008), dealing with the care and treatment of patients who either are unable to give their consent or are opposed to receiving necessary help and treatment. A commentary on this law states that various diseases and conditions arising during diseases could negatively impact patients’ competence and, in so doing, prevent patients from giving consent. The law ensures that necessary health care is provided even if patients do not understand its implications or consequences. The nurses interviewed for this study said that patients suffering from COPD are often not in their full senses and are not able to participate in decisions about their treatment. A circular from the Norwegian Board of Health Supervision stated that ‘medicine can be administered with sedative and anaesthetic purposes, if this is considered necessary to carry out effective health help’ (p. 35 § 4 A-4, 3.4.2).

In certain respects, the discourse of patient participation expressed in these interviews does not correspond with the way in which this concept is used both in various Norwegian and in international documents and literature (WHO 1994, White paper no. 25 2005–2006, Eldh 2006, Sahlsten *et al.* 2008). Definitions, declarations, and regulations are often expressions of democratic ideals – and in this context, a level of patient participation that is desired. In White paper no. 25 (2005–2006) to the Norwegian Parliament, the government does not discuss patient participation that is marked by non-involvement. The nurses interviewed for this study explained that they indirectly try to increase patient participation. For example, they read their patients’ body language and attempt to establish a joint understanding with their patients of how mask treatment should be administered. The nurses also revealed that they consult the next of kin when their patients do not have the capacity to express information verbally. Our findings are in agreement with those made by Traynor *et al.* (2010), who found that nurses sometimes use indirect means to exercise influence in a clinical and bureaucratic setting. Despite limited autonomy, nurses told stories of their successful performance of moral and influential actions.

The nurses interviewed for the present study said that they were preoccupied with building trust and exuding confidence when interacting with patients suffering from COPD. Emotional reciprocity was also considered important in these situations. They tried to meet the suitable levels of involvement for individual patients and their situations. In agreement with Thompson *et al.* (2007), the nurses in this study showed awareness of the variations in participation according to the different phases of illness.

The participating nurses also discussed the difficult ethical situations that occur when the decision to administer palliative or curative treatment to a patient in the last stages of COPD has not been made. Gardiner *et al.* (2010) identified a clear need for pragmatic strategies to help enhance communication and information sharing in treating patients with COPD. When healthcare personnel decline to meet challenges in this area, patients can be prevented from receiving palliative treatment. Hence, close cooperation between nurses and physicians is imperative when treating patients with COPD.

**What is already known about this topic**

- Patients often experience chronic obstructive pulmonary disease exacerbation to be extremely frightening.
- Patient participation is a predominant issue in health care in many countries worldwide.
- The concept of patient participation is complex and multifactorial in nature.

**What this paper adds**

- Care in the acute phase of chronic obstructive pulmonary disease is often characterized by low level of patient power and participation.
- Nurses consider emotional reciprocity to be important.
- Treatment in acute phases involves dealing with challenging ethical situations.

**Implications for practice and/or policy**

- Nurses must develop skill, understanding, and competence to meet ethically difficult care situations that often occur during an exacerbation.
- A collaborative inter-professional approach between physicians and nurses is needed to meet the patients’ demand for involvement during exacerbation.
- Patient participation should be understood as a dynamic two-way communication process.

All of the focus groups interviewed for the present study agreed that the acute phase was not the appropriate time to discuss palliative versus curative treatment. However, even in cases where patients and their healthcare team have discussed the issues and decisions have been made in advance regarding patient care, challenging ethical issues may still arise. For example, a nurse interviewed for the present study described a situation in which a patient close to death suddenly begged for ventilator treatment, although it had previously been decided that he should receive only palliative treatment. Previous studies have shown that nurses in intensive care units experience stress induced by ‘dissonant imperatives’ (Cronqvist *et al.* 2001, Mackintosh 2006). In the present study, the nurses were frustrated when no experienced physicians were present to make decisions in critical situations. Lacking the authority to act and at the same time knowing that something should be done seemed to be particularly stressful for the nurses. Oberle and Hughes (2001) point to the need for increased dialogue within and between medicine and nursing around the ethical aspects in end-of-life decisions. This study has also shown the need for a cross-disciplinary discussion of ethical dilemmas in COPD exacerbation.

### Study limitations

This study had some limitations. First, patient participation was viewed only from the perspective of experienced intensive care nurses. It would be of great interest to obtain information from other perspectives (e.g. the perspectives of patients, next of kin and physicians) so as to understand more fully the special challenges involved in patient participation during the acute phase of COPD exacerbation. Second, the individual (the first author of this article) who was responsible for selecting samples for detailed analysis and for describing the data was an intensive care nurse, which may imply that she was too closely affiliated with the field to interpret the data impartially. Researchers with different backgrounds might find other themes than those presented here.

## Conclusion

This study has shown that according to intensive care nurses, it can be difficult to take care of patients’ rights to participate during COPD exacerbation. Patient participation should be understood as a dynamic two-way communication process. One way to achieve participation during the acute phases could be to let the patients get a contact nurse in the intensive care unit who discusses with the patients what kind of care the patients wish during the next exacerbation. A collaborative inter-professional approach between physicians and nurses is needed to meet the patients’ demand for involvement. Patient participation should be an important theme in the curriculum in nursing education and in medical education. Further research on how patients and next of kin view participation under these extreme circumstances is needed to get a deeper understanding of this field.
